# Supplementary material for: Comprehensive Annotation and Functional Exploration of MicroRNAs in Lettuce
Source: Front Plant Sci. 2021 Dec 24;12:781836. doi: 10.3389/fpls.2021.781836 (PMC8739914; doi:10.3389/fpls.2021.781836)
Supplement: Supplementary file 2 [file Data_Sheet_8.ZIP › GSM2230748.plot/Lsa-miR397_Lsat_1_v5_gn_9_24581.1_742_TPlot.pdf]

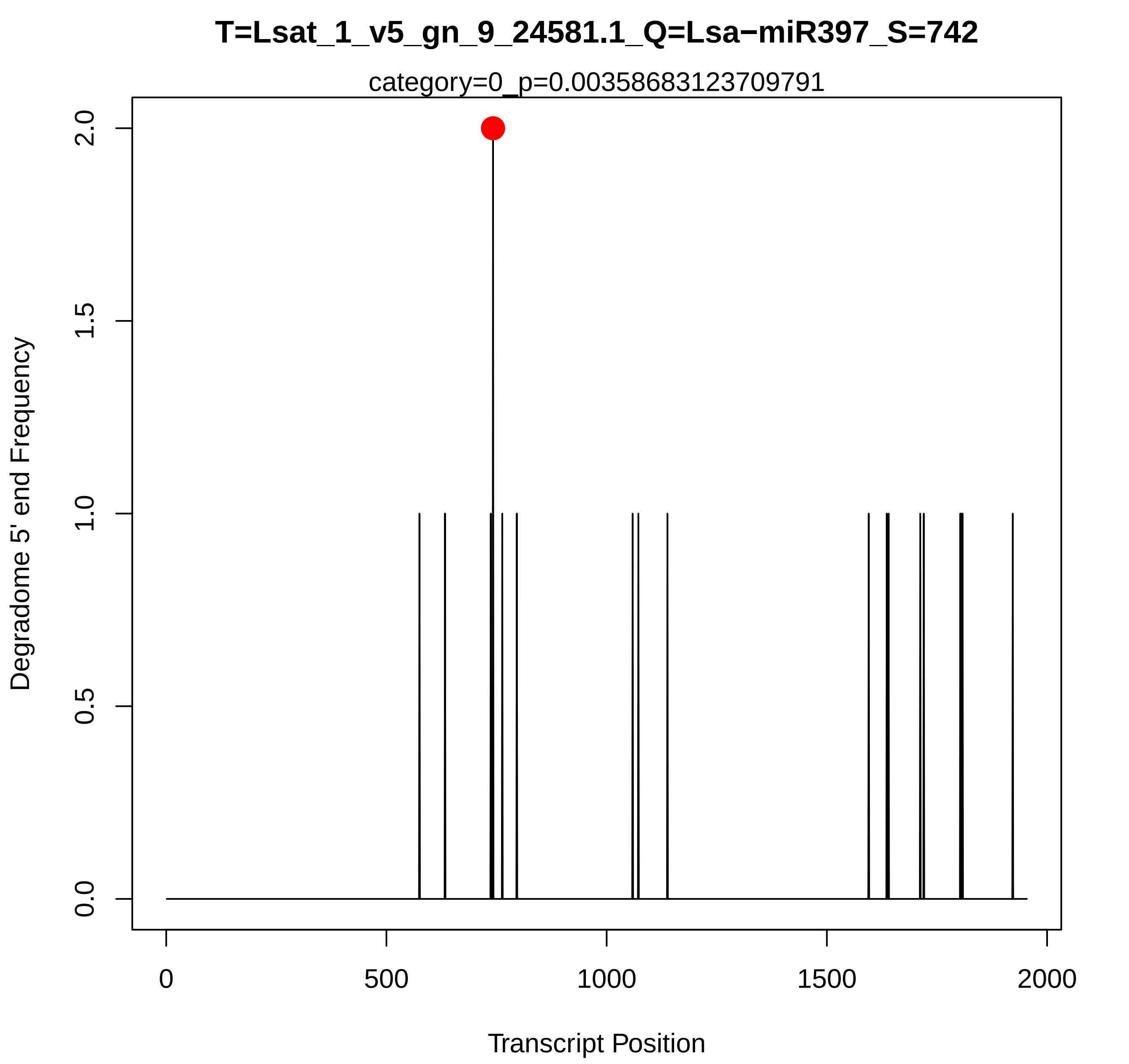

T=Lsat_1_v5_gn_9_24581.1_Q=Lsa−miR397_S=742
category=0_p=0.00358683123709791
Degradome 5' end Frequency
0.0
0.5
1.0
1.5
2.0
0
500
1000
1500
2000
Transcript Position
